# A *FBN1* variant manifesting as non-syndromic ectopia lentis with retinal detachment: clinical and genetic characteristics

**DOI:** 10.1038/s41433-019-0580-2

**Published:** 2019-09-16

**Authors:** Kirk A. J. Stephenson, Adrian Dockery, Michael O’Keefe, Andrew Green, G. Jane Farrar, David J. Keegan

**Affiliations:** 1 Retinal Research Group: Mater Misericordiae University Hospital & Mater Private Hospital, Dublin, Ireland; 2 School of Genetics and Microbiology, Trinity College Dublin, Dublin, Ireland; 3 National Children’s Research Centre, Our Lady’s Children’s Hospital, Crumlin, Dublin, Ireland

**Keywords:** Hereditary eye disease, Lens diseases, Retinal diseases, Eye manifestations, Disease genetics

## Abstract

**Background/objectives:**

Fibrillin-1 (*FBN1*) mutations cause connective tissue dysgenesis the main ocular manifestation being ectopia lentis (EL), which may be syndromic or non-syndromic. We describe a pedigree with a *FBN1* mutation causing non-syndromic EL with retinal detachment (RRD) and their management.

**Subjects/methods:**

Patients with familial EL with RRD were invited to participate (vitreoretinopathy branch of Target 5000, the Irish inherited retinal degeneration study). All patients signed full informed consent. The study was approved by the Institutional Review Board of the Mater Hospital, Dublin and abided by the Declaration of Helsinki.

**Results:**

Seven adults were affected with bilateral EL. All subjects had RRD with bilateral non-synchronous RRD in 57%.

**Conclusions:**

The *FBN1* variant described herein confers an increased risk of both EL and RRD and can now be upgraded to ‘pathogenic’ ACMG status.

## Background

Mutations in the fibrillin-1 gene (*FBN1*, chromosome 15q21.1, OMIM *134797) are associated with autosomal dominant disorders of connective tissue. Ectopia lentis (EL) is the primary ocular manifestation of the type-1 fibrillinopathies that include non-syndromic EL (NSEL) (OMIM #129600) and syndromic EL (SEL). The phenotypic manifestation of SEL versus NSEL and their severity depends on the position and extent of pathogenicity of the causative *FBN1* mutation.

*FBN1* is a large gene (>200 kb) comprising 66 exons, producing a five domain, 2871-amino acid extracellular protein [1]. Mutant fibrillin protein disrupts microfibril formation and function [2] degrading more rapidly than wild type [3–5]. TGF-β-mediated inflammatory elastolysis may play a role [6]. Proteomic studies indicate fibrillin-1 is the most abundant protein in ciliary zonules [7, 8]. Knockout of *FBN1* in a murine model manifested zonular rupture [9].

The archetypal type-1 fibrillinopathy is Marfan Syndrome (MFS, OMIM #154700) whose diagnostic criteria include cardiac (aortic dissection), ocular (EL) and systemic signs (catalogued in the revised Ghent Criteria [10]). The major ophthalmic criterion is EL. However, other ocular features include axial myopia, corneal flattening, astigmatism, glaucoma and rhegmatogenous retinal detachment (RRD) [6, 11, 12]. Phenotype may be exclusively ocular (i.e. NSEL), or include a systemic syndrome (SEL), relevant for prognosis/management.

## Methods

Patients with EL were recruited to Target 5000 [13–15]. All participants completed written informed consent. This study was approved by the institutional review board of the Mater Hospitals, Dublin, Ireland and abides by the Declaration of Helsinki.

Patients underwent a detailed history and comprehensive ophthalmic examination. Affected relatives were included. Relevant historical ophthalmic details were included and retrospective non-randomised, non-masked analysis was performed. Multimodal retinal imaging was performed: colour and autofluorescence imaging (Optos plc, Scotland) and Optical Coherence Tomography (SD-OCT, Carl Zeiss Meditec, Germany).

Genotyping by direct sequencing was performed at The School of Genetics and Microbiology, Ocular Genetics Lab, Trinity College Dublin. The variant described herein was first observed in index cases by the Wessex Regional Genetic Laboratory.

The surrounding genomic region was amplified by polymerase chain reaction (PCR, primers: forward: 5′-CCCTGTTGGTTTGTTGCTCT-3′; reverse: 5′-TGAGAATGCCATTTGAGCTTTTG-3′). Templates were amplified using Q5 High Fidelity Polymerase (New England Biolabs). PCR products were purified using the GeneJET Gel Extraction Kit (Thermo Fisher Scientific). Sanger sequencing was conducted by Eurofins Genomics (Ebersberg, Germany).

## Results

**Fig. 1:**
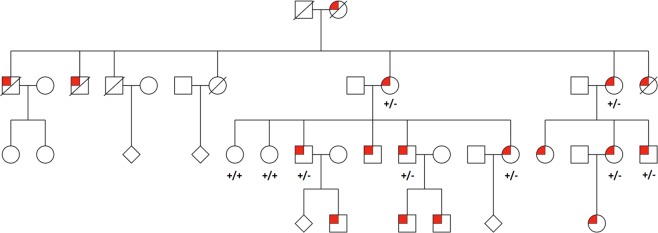
Pedigree diagram of non-syndromic ectopia lentis showing autosomal dominant inheritance. All investigated individuals are annotated with their observed genotype concerning the variant *FBN1* NM_000138:c.1916G>A, p.Cys639Tyr. A ‘plus’ indicates an agreement with the reference base whereas a ‘minus’ indicates the presence of the variant of interest. Squares, circles and diamonds represent males, females and people of unknown gender, respectively. Individuals affected by ectopia lentis are indicated by a red marker

Seven affected (mean 58.43 ± 15.02 years) and two unaffected (mean 56.50 ± 0.71 years) adults from two generations of a single NSEL pedigree were recruited (Fig. 1). Those included had bilateral EL, high axial myopia (axial length (AL) 26.66 ± 1.29 mm, *n* = 10/14) and RRD/intraoperative breaks) without other trauma.

RRD was diagnosed in all seven affected patients (11/14 eyes, 78%) and zero eyes of unaffected relatives. Bilateral non-synchronous RRD occurred in 4/7 affected subjects (57%). Re-detachment occurred in 1/11 (9%). RRD occurred intra-/post-operative in 64%, the remainder being surgically naïve eyes. The RRD rate in this pedigree is significantly higher than the reported rate of EL-related RRD, (0–25%) [6, 11, 16–20].

There was no personal/family history of aortic root/valve defects confirmed by normal serial echocardiography. Thus, no member of this pedigree meets the revised Ghent criteria for the diagnosis of MFS [10].

Molecular genetic testing detected a previously reported [21] mutation in *FBN1* [1] (NM_000138:c.1916G>A, p.Cys639Tyr) although detailed phenotyping was not available in the prior study. In the current study, the Cys639Tyr mutation was confirmed in all affected adults and neither unaffected relative.

## Discussion

### Genetics

Structurally relevant *FBN1* variants are seen in SEL including MFS [22], but also in NSEL [21, 23]. *FBN1* mutations are the primary cause of inherited EL (SEL and NSEL), although other genes have been implicated in recessive EL (e.g. ADAMTSL4, ADAMTS10) [21, 24]. Most EL pedigrees have private *FBN1* mutations [25] (>1500 variants reported [26–28]). This same rare *FBN1* variant manifesting in two disparate ethnic groups suggests sensitivity to mutagenesis at this position, a postulation supported by four different pathogenic missense variants reported in p.Cys639 [29].

This missense variant replaces a cysteine residue with tyrosine in one of the fibrillin-1 protein’s EGF-like domains. Fibrillin-1 cysteine loss is associated with zonular instability/EL [19, 30, 31]. It is plausible that this pedigree lacks any additional pathogenic cardiac risk variants although comprehensive testing of these genes (e.g. *SMAD3, COL4A1, ECE1*) was not performed. The c.1916G>A variant has been previously reported in segregation with NSEL [21]; however, detailed phenotyping was lacking and ACMG grading only satisfied the ‘likely pathogenic’ variant status criteria. Detailed phenotyping and lines of mutation evidence from this pedigree allows promotion of this *FBN1* variant to ‘pathogenic’ ACMG status.

### Systemic

The fatal manifestation of MFS is aortic dissection, typically occurring before 40 years [32] (mean age of affected individuals here being 58.43 ± 15.02 years). Aortic root disease in type-1 fibrillinopathies may be progressive, thus NSEL should not be diagnosed before 20 years of age and serial echocardiography is recommended for life [10, 23].

We have referred to this pedigree’s phenotype as ‘NSEL’ to dissociate both from MFS stigma (i.e. insurance, mortality [10]) and to accentuate the other blinding ocular features that ‘isolated EL’ does not adequately highlight (i.e. RRD, glaucoma). Presentation with poor vision often occurs early in both MFS and NSEL [21] allowing detection of both familial and sporadic cases, facilitating instigation of systemic investigations and treatment.

Axial myopia may be (1) a genetic feature of this *FBN1* variant, (2) related to other myopia risk genes or (3) acquired as a result of lens blur-induced myopia from the ectopic lens [33]. Longer AL is associated with both higher prevalence of EL and RRD in type-1 fibrillinopathies [34].

RRD was diagnosed in all affected patients, with 57% bilateral, non-synchronous RRD. In this cohort intraoperative retinal tears were documented in 14% (*n* = 2/14), a feature not reported in previous EL publications. Thirty-six percent of RRD (*n* = 4/11) occurred in surgically naïve eyes with no precipitating trauma. Individual surgeon factors can be excluded from the remainder as surgery was performed by three separate vitreoretinal surgeons. In large cohorts of SEL (mainly MFS), RRD is a prominent feature both pre- and post- vitreolensectomy surgery [11], the published post-operative RRD rate ranging from 0 to 25% [3, 6, 10, 11, 16–21, 27, 28]. Thus, this *FBN1* variant may confer an increased risk of RRD (100% of individuals, 79% of eyes) and intraoperative retinal breaks. There is a lack of data in the literature on the retinal phenotype of NSEL. This may be due to an automatic labelling of any case of EL as ‘MFS.’ Prophylactic 360-degree retinopexy akin to the Stickler Syndrome Cambridge protocol [35] may be performed.

The current study investigates the phenotype of a single pedigree and a single *FBN1* variant whereas, other studies describe various unrelated EL probands with distinct *FBN1* variants. This may partly explain the high incidence of RRD in this cohort. Regular surveillance for RRD must be performed for all people with a known pathogenic *FBN1* mutation or an EL phenotype whether syndromic or not.

## Conclusion

The genetic findings from this pedigree add significantly to the existing data, upgrading the ACMG grading of this *FBN1* variant to pathogenic. The ability to perform targeted sequence-based testing for this specific *FBN1* variant in future generations will allow informed decisions to be made regarding necessity and timing of intervention/prophylaxis [36] for EL and RRD.

## Summary

This paper describes a pedigree with a pathogenic *FBN1* variant manifesting as non-syndromic ectopia lentis with prevalent retinal detachment. Systemic features (specifically cardiac) were absent in all cases.

### What was known before

- Ectopia lentis is a feature of Marfan Syndrome, difficult refractive management choices, especially in children.
- Retinal detachment is a common feature (up to 25% of cases).

### What this study adds

- A specific missense variant with 100% of affected individuals with detachment, 57% bilateral. Genetic diagnosis, monitoring critical ± prophylaxis.
